# Use of Technology in an Internet-Delivered Intervention for Older Adults With Mild Cognitive Impairment

**DOI:** 10.1093/geroni/igaa057.2725

**Published:** 2020-12-16

**Authors:** Meghan Mattos, Laura Barnes, Eric Davis, Carol Manning, Mark Quigg, Lee Ritterband

**Affiliations:** 1 University of Virginia, Charlottesville, Virginia, United States; 2 University of Virginia, School of Medicine, Charlottesville, Virginia, United States

## Abstract

Internet-based interventions using technology can promote access to treatment and reduce participant burden for sleep disorders. However, preliminary studies examining technology use and compliance in older adults with mild cognitive impairment (MCI) are needed prior to undertaking large-scale interventions. Older adults with MCI were recruited from hospital-based memory and sleep disorders clinics and enrolled in a single-arm intervention pilot study. An Internet-delivered cognitive behavioral therapy for insomnia program collected daily sleep diary data and delivered the automated intervention over nine weeks. Sleep diaries and wrist-worn actigraphs collected sleep data for 14 days, pre- and post-intervention. Descriptive statistics for participant technology use are presented. We have recruited 12 subjects with MCI. Most subjects with MCI accessed the intervention program daily; however, actiwatch compliance varied. Incorporating technology for intervention delivery and data collection in this population is promising, and future work should consider using reminders with wearable technology to increase compliance.

